# Impact Absorption Behaviour of 3D-Printed Lattice Structures for Sportswear Applications

**DOI:** 10.3390/polym17192611

**Published:** 2025-09-26

**Authors:** Mei-ki Chan, Sik-cheung Hung, Kit-lun Yick, Yue Sun, Joanne Yip, Sun-pui Ng

**Affiliations:** 1 School of Fashion and Textiles, The Hong Kong Polytechnic University, Hong Kong; meiki-0125.chan@connect.polyu.hk (M.-k.C.); sikcheung.hung@connect.polyu.hk (S.-c.H.); mailtcjyip@polyu.edu.hk (J.Y.); 2 School of Fashion Design and Engineering, Zhejiang Sci-Tech University, Hangzhou 310018, China; sunyue@zstu.edu.cn; 3 School of Professional Education and Executive Development, The Hong Kong Polytechnic University, Hong Kong; zerance.ng@cpce-polyu.edu.hk

**Keywords:** sports protective equipment, lattice structures, additive manufacturing, energy absorption, force reduction, re-entrant, rhombic dodecahedron

## Abstract

Lattice structures have been widely studied in various fields due to their lightweight and high-energy absorption capabilities. In this study, we propose the use of lattice structures in the design of sports protective equipment for contact sports athletes. A total of six specimens were additively manufactured either with a bending-dominated rhombic dodecahedron (RD) structure or stretch-dominated re-entrant (RE) structure. Elastic resin was used to investigate the specimens’ compressive strength and energy absorption, impact reduction, and flexural properties in comparison with those of conventional foam and rigid polyethylene (PU). Despite having a lower relative density, the RE structure exhibits greater stiffness, showing up to 40% greater hardness and averaging 30.5% higher bending rigidity compared with the RD structure. However, it unexpectedly shows less stability and strength under uniaxial loading, which is 3 to 6 times weaker when compared with the non-auxetic RD structure. Although conventional PU has higher loading than 3D-printed lattices, the lattice shows excellent bendability, which is only 1.5 to 3 times stiffer than that of foam. The 3D-printed lattice in this study shows an optimal improvement of 43% in terms of impact absorption compared with foam and a 2.3% improvement compared with PU. Amongst the six different unit cell dimensions and structures studied, the RD lattice with a cell size of 5 mm is the most promising candidate; it has superior elasticity, compressive strength, and impact resistance performance whether it is under low- or high-impact conditions. The findings of this study provide a basis for the development of 3D-printed lattice sports protective chest equipment, which is more comfortable and offers improved protection for contact sports players.

## 1. Introduction

Contact or collision sports, such as rugby, American football, hockey, baseball, and basketball, are athletic activities characterised by significant physical interactions between players, the environment, and equipment, which involve tackles, collisions, and other forms of direct contact [1]. Around 60% of contact sports injuries are caused by physical collisions and tackles [2]. It has been reported that around 136 collisions occur in a professional rugby league match [3]. High-intensity body collisions, involving movements that generate significant force, momentum, and changes in direction, inherently carry a higher risk of injuries, such as fractures, concussions, musculoskeletal injuries, and commotio cordis, in comparison with non-collision sports like soccer and cricket [2,4,5,6]. As the number of sports enthusiasts continues to rise, there is a corresponding increase in the demand for sports protective gear, accompanied by higher expectations for the quality of these products. Helmets, support sleeves, shin guards, shoulder guards, and other padded chest or shoulder gear are common types of protective devices and equipment used in many sports to protect soft tissue, joints, and bones against impact [7].

Despite the known risks, the usage of protective gear, especially chest gear, in contact sports has been largely ignored [7]. This can be attributed to several factors, including the perception that protective equipment may hinder performance by adding weight or restricting movement, that it can be uncomfortable to wear, and that its protection effectiveness can vary [8]. Sports protective equipment for contact sports players is mainly made of moulded plastic or high-density foam like ethylene-vinyl acetate (EVA) and polyurethane (PU) [9]. Composed of rigid and impermeable materials, although impact protectors are meant to keep athletes safe, their capacity to absorb shocks frequently conflicts with their comfort, weight, and range of motion, which is also the main reason for the low usage of chest protective equipment [8,10]. Additionally, some athletes may underestimate the risk of injury or prioritise comfort and agility over safety. There is also a cultural aspect, where toughness and resilience are often emphasised, leading to a reluctance to adopt comprehensive protective measures. According to the National Operating Committee on Standard for Athletic Equipment (NOCSAE) standard, chest protectors for baseball or lacrosse players should be able to reduce impact to below 400N or 800N under 30 or 50 mile per hour conditions so as to prevent commotio cordis [5]. In addition, it is emphasised that athletes’ typical movements should not be restricted [8]. This also highlights the importance of ensuring that sports protective equipment fits correctly and that players conform to using it. As a result, there is a critical need for innovative solutions, like lattice structures, that can provide effective protection without compromising the athletes’ performance or comfort to enhance sports protective equipment usage and guarantee athletic performance.

Lattice structures are architectures composed of a series of interconnected repeating unit cells with edges and faces, which can be in both two and three dimensions [11]. Characterised by a repeating unit cell, lattices are engineered to have unique mechanical properties, such as being lightweight and having a high strength-to-weight ratio, which are advantageous for energy absorption and impact resistance. Also, lattices have been widely applied in varied industries, such as aerospace [12] and healthcare [13]. The mechanical properties of lattice structures are highly affected by cell topology, relative density, and base material [14], which can mainly be divided into two extremes, namely stretch-dominated and bending-dominated topologies, according to their deformation behaviour. Re-entrant cellular structures are common types of stretch-dominated auxetic structures that have been used in sports protective gear, energy-absorbing systems, and prosthesis [15,16,17] due to their unique behaviour, i.e., that they experience transverse shrinkage while compressed and expand when stretched, resulting in a negative Poisson’s ratio that exhibits higher strength and stiffness. Lattices are commonly used in lightweight and load-carrying applications [18,19], and they can offer a reduction in peak transmitted pressure; therefore, they can be effectively used for blast damage reduction [17]. Many studies have focused on the structural effect of cell angles and sizes of 3D-printed re-entrant structures in terms of impact resistance performance, especially in helmets [20,21,22,23]. On the other hand, bending-dominated lattice structures are mainly used for energy absorption applications due to their compliance characteristics, where the compressive load is distributed by the bending and fracture of the struts [24,25]. The mechanical performance of the rhombic dodecahedron structure has been widely studied, especially for cushioning and shock-absorbing applications such as helmet liners [26], robotic foot [27], and the replacement of dense implants [28], due to its high energy absorption, high biological compatibility, and low shear stiffness [29]. Zhang and Kong et al. [27] found that a robotic foot pad designed with a rhombic dodecahedron structure can reduce impact force by 82% and reduce force from 302.1 kN to 54.5 kN compared with a solid foot pad. The structure’s outstanding energy absorption performance is expected to offer more effective force protection while maintaining comfort through its lightweight design compared with solid force-absorbing materials. Advancements in additive manufacturing (AM) technology allow for the fabrication of more complex structures with specific strengths and functions and with a wider variety of materials that could not be produced by conventional manufacturing processes.

Over the years, a number of studies have been performed regarding the mechanical performances of typical or modified 2D and 3D auxetic and lattice structures, such as graded effect and multi-structural design, in terms of compressive behaviour [30], quasi-static strength [31,32], shear properties [33], and flexural performance [34]. However, the analysis of impact reduction behaviour has been neglected. Furthermore, most research has focused on metallic, concrete, stiff, or brittle materials, such as stainless steel and aluminium foams [19,35,36]. Limited research has been performed regarding the application of lattice structures in elastic materials and wearables like chest gear. This study focuses on investigating the unit cell size effect and mechanical properties of a 3D stretch-dominant re-entrant lattice structure and a bending-dominated open-cell rhombic dodecahedron cellular structure in terms of bending elasticity, compressive behaviour, and force reduction property. The performance of the lattice structures was compared with that of conventional materials of breast protective equipment to analyse the feasibility of a 3D-printed chest protector.

## 2. Materials and Methods

### 2.1. Lattice Design and Production

**Figure 1:**
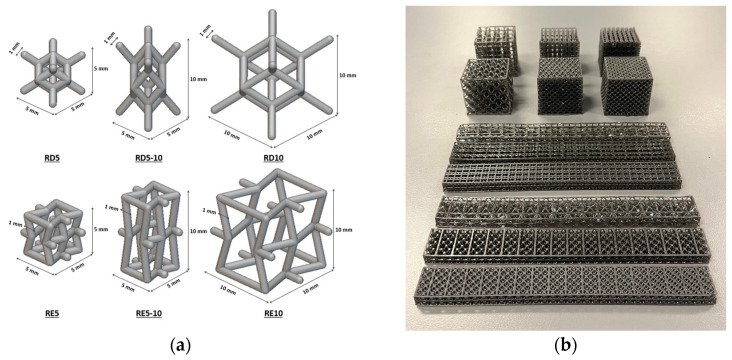
(**a**) Dimensions of the RD and RE unit cell structure; (**b**) 3D-printed specimens for compression and bending test.

Two conventional materials used for sports chest protectors, including rigid polyethylene (PU) (MaxiGuard, QP sports, New Zealand) and flexible closed-cell foam (sourced from TaoBao), were used in this study. Both rhombic dodecahedron and re-entrant structures with three different unit cell sizes were characterised. The maximum unit cell size was set to 10 mm to match the thickness of conventional chest protection equipment. The geometry and dimensions of the samples are shown in Figure 1a, featuring unit sizes of 5 × 5 × 5 mm^3^, 5 × 5 × 10 mm^3^, and 10 × 10 × 10 mm^3^, resulting in a unit cell ratio of 8:4:1. The aspect ratio was determined by the unit cell dimensions, calculated as height/width. Using nTopology software, samples were printed in various dimensions to meet the standard test requirements. With a constant wall thickness of 1 mm, the volume and mass of the structures varied according to cell size. The relative density (ρ) was calculated from the measured weight of the samples using the ratio of the lattice material (ρ*) to the bulk material density (ρS), i.e., ρ*/ρS [37]. Table 1 lists the measured mass and relative density of the 40 × 40 × 40 mm^3^ lattice samples.

Supporting structures were added to the edges of each sample in order to improve the printing of the samples and their stability. The samples were then printed using a 3D printer LuxCreo 3Li+ (LuxCreo Inc., Chicago, IL, USA), which is based on Digital Light Processing (DLP) technology. A flexible resin EM+23 (LuxCreo Inc., Chicago, IL, USA) with a shore hardness of 70A and a density of 1.01 g/cm3 (MAT #EM232101-01V1.1) was used for the printing. Detailed mechanical properties are summarised in Table 2. The material was preheated to 40 °C and then printed with 100% infill density and 0.16 mm layer thickness with a light intensity of 3400 uw. After printing, the parts were washed, spin-dried to remove excess resin, and then thermally cured in an oven. The printed samples are shown in Figure 1b.

### 2.2. Experimental

The mechanical performances of lattice structures for impact-protective sportswear were investigated in this study. Compression, drop, and bending tests were conducted to assess the impact protection ability, deformation pattern, compressive behaviour, and 3D shape conformity of the proposed lattice structures. The results were systematically compared with those of conventional foam insert and PU plates in protective sportswear.

#### 2.2.1. Compression

**Figure 2:**
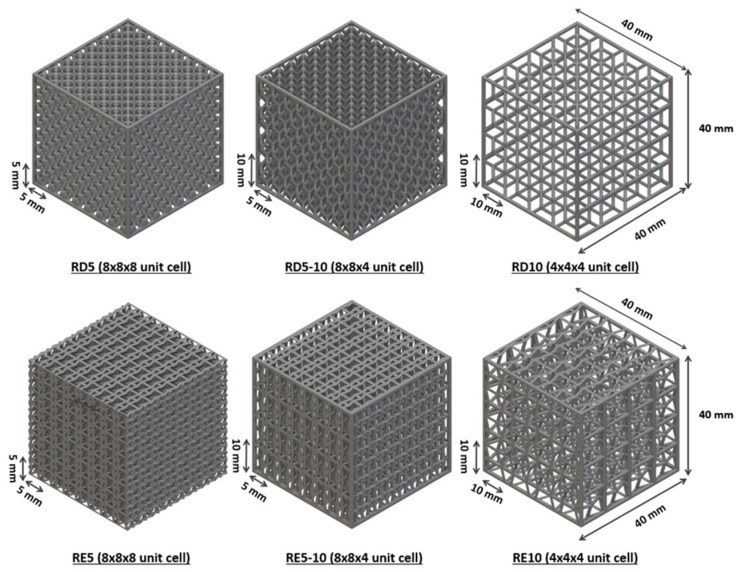
Dimensions of the RD and RE lattice specimens with varied unit cell sizes for the compression test.

A quasi-static uniaxial compression test according to BS EN ISO 3386-2:1997 (polymeric materials [38], which are cellular and flexible, and determination of stress–strain characteristics in compression) was performed to analyse the deformation and compressive strength of the conventional material and proposed structures. The Instron 5566 (Norwood, MA, USA) universal mechanical test frame is equipped with a 10 kN load cell. Three samples were prepared for each specimen. The samples were compressed with a constant deformation rate of 10 mm/min in the Z-direction to 40% of their original height (i.e., 16 mm) for four loading and unloading cycles. Three specimens for each structure with nominal dimensions of 40 mm (L) × 40 mm (W) × 40 mm (H) were prepared for the compressive test (Figure 2). The compressive stress value (CV) was calculated based on the following equation:

CV = 1000 × F40/A,(1)

where F40 is the force (N) at 40% compression at the fourth loading cycle, while A is the surface area (mm) of the test sample.

The energy absorption capacity (EA) is calculated per unit mass, as presented in Equation (2):

EA = E/m(2)

where E is the total energy absorbed (J), and m is the mass of the sample (kg).

Poisson’s ratio was calculated by measuring the change in unit cell size in the longitudinal and transverse dimensions using a digital camera based on the following equation:

*ν* = (−ε_trans_)/ε_long_ (3)

where *ν* is Poisson’s ratio, ε_trans_ is the transverse strain, and ε_long_ is the longitudinal strain.

#### 2.2.2. Impact Force Reduction

**Figure 3:**
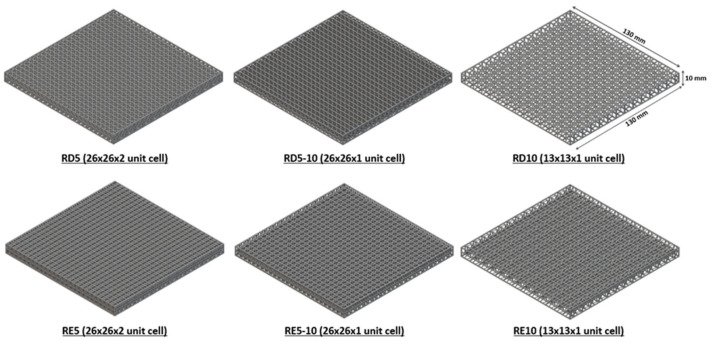
Dimensions of the RD and RE lattice specimens with varied unit cell sizes for ball drop test.

According to the NOCSAE (National Operating Committee on Standards for Athletic Equipment) standard ND200-22, chest protectors for baseball and softball must reduce the impact peak force from approximately 1414 N to ≤400 N at 30 mph impacts and from 2685 N to ≤800 N at 50 mph impacts [39]. This requires a reduction of over 70% in impact force for both low- and high-velocity conditions. To evaluate this, a ball drop test (ASTM D2632-15) was performed using ball weights of 45.9 g and 260 g [40], which were dropped from a height of 400 mm, with an average peak force of 500N and 3300N, respectively. The test assessed the force reduction performance of six proposed structures and conventional samples under varying impact forces. Specimens with a dimension of 130 mm × 130 mm × 10 mm (Figure 3) were placed at the bottom of a vertical tube, and the impact force was measured using a Dytran force sensor (1051V5, IEPE, Dytran, CA, USA) with a sensitivity of 5.13 mV/Lb.F. Each test was repeated ten times to determine the force reduction capability of the materials using the following equation:

FRx = (1 − (Fx/F0)) × 100%(4)

where FRx is the percentage of impact force redacted (%), and Fx is the measured peak force of the sample (N), while F0 is the measured peak force of the ground surface (N).

#### 2.2.3. Bending Test

The three-point bending test, following ASTM D790-17 [41], evaluates the elasticity and flexural properties of the test samples. Using a Universal machine Instron 5566 with a 50N load cell, three specimens with dimensions of 176 mm × 20 mm × 10 mm were prepared for each structure. Each specimen was placed on two support rods with a 100 mm span, and a load was applied at the midpoint at a speed of 10 mm/min to create a bending force. This test measures the material’s ability to withstand bending stress, assessing its flexibility, strength, and elasticity. These properties ensure that the impact resistance materials fit and conform to the body contour of the wearers. The modulus of elasticity is presented as follows:

Eв = L3F/4bd3(5)

where Eв is the modulus of elasticity (GPa), L is the length of the support span (100 mm), F is the maximum applied stress at the midpoint (kPa), b is the width of the specimen (20 mm), and d is the depth of the specimen (10 mm).

## 3. Results and Discussion

### 3.1. Compressive Behaviour

**Figure 4:**
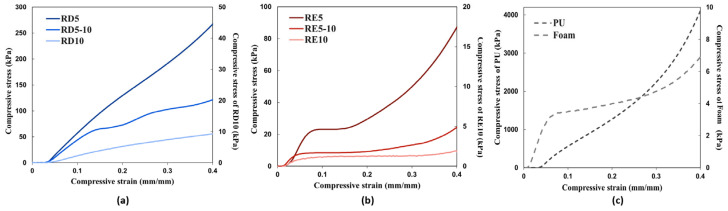
Stress–strain response comparison within (**a**) RD structure, (**b**) RE structure, and (**c**) conventional foam and PU.

**Figure 5:**
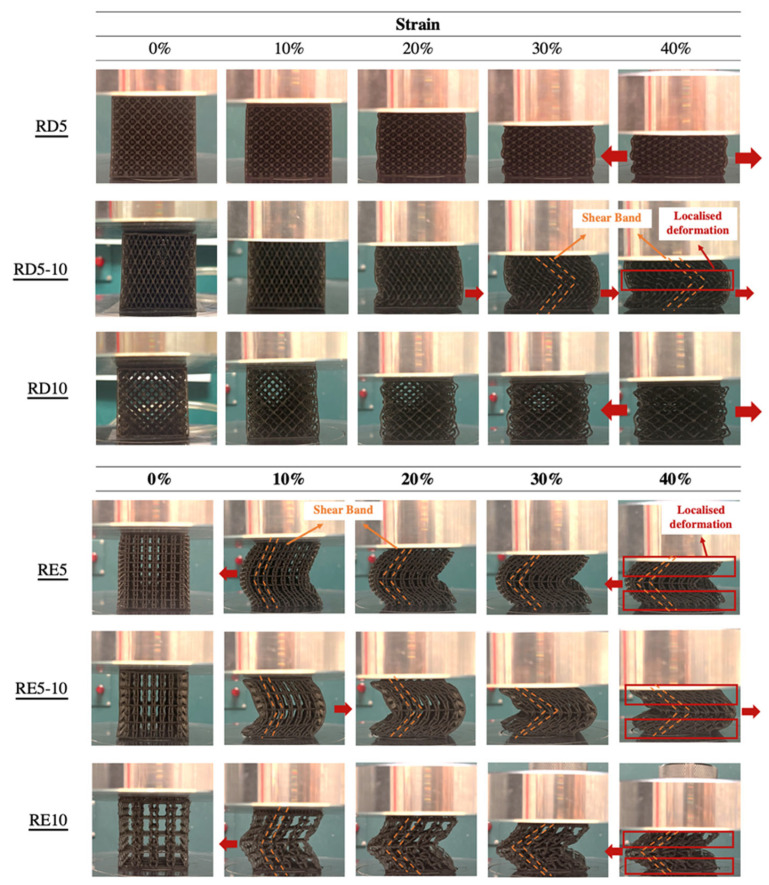
Deformation of RD and RE specimens at 10, 20, 30, and 40% strain.

Figure 4 and Figure 5 demonstrate the stress–strain curves and deformation of the three RD- and RE-based lattice structures with varying cell sizes, as well as the conventional foam and PU materials at the fourth loading cycle. The RD structures show a significantly stronger compressive strength than the RE samples, of 167-494%, while there is no significant negative Poisson’s effect among the auxetic RE structures. According to the stress–strain curves shown in Figure 4a, the RD structures mostly exhibit a linear elasticity behaviour. This response can be attributed to their highly symmetrical configuration, characterised by an interconnected network in which each face forms a rhombus [13], resulting in layer-by-layer uniaxial deformation. This distinctive attribute facilitates the uniform distribution of compressive forces across multiple beams, thereby minimising localised stress concentrations through bending deflection. Nevertheless, with an aspect ratio of 2, RD5-10 shows two distinct peak stresses at around 0.15 and 0.25 mm/min strain. Contrary to McDonnell et al.’s findings [42], an increase in aspect ratio does not correlate with enhanced mechanical performance when compared with uniform geometry (RD5). Unlike the rigid material employed in the previous study (17-4PH stainless steel, UTS (tensile) = 1103 MPa), the elastic material used in this research (EM+23, UTS (tensile) = 21.59 MPa) demonstrates a greater deformation range before it reaches failure. A higher aspect ratio may result in a higher risk of local buckling, thus reducing the stiffness and compressive strength in the bending-dominated lattice [43].

On the other hand, localised deformation and the shear band were observed among all the RE-based structures (Figure 5). Different from the linear stress–strain curve of the RD structures, the RE structures demonstrated three deformation stages, including the linear–elastic stage, plastic deformation, and the compacting stage [44], which is similar to the stress–strain response of the foam sample (Figure 4b,c). The compressive strength of the structure significantly dropped as the aspect ratio increased. According to Choi and Park [45], Poisson’s ratio of the auxetic re-entrant structure ranges between −0.3 and −0.5. However, a positive value was obtained for RE5 (Table 3). In contrast, increasing the cell sizes (RE5-10 and RE10) resulted in a transition to negative Poisson’s ratios. It is anticipated that RE5 does not fully exhibit auxetic behaviour, resulting in a small positive value. While the cell size increases (RE5-10 and RE10), the structure may more readily deform in an auxetic manner, leading to more negative Poisson’s ratios. Nevertheless, the force may also exceed the elastic limit of the material that alters the expected Poisson’s ratio, leading to the local buckling and collapse of auxetic cells; thus, there is a more nonuniform stress distribution across the lattice and poorer load-bearing capacity.

In terms of energy absorption, the bending-dominated RD structures show larger energy absorption per mass than the stretch-dominant RE structures (Table 3). The RD and RE structures exhibit distinct deformation mechanisms, where the former deforms through bending, whereas the latter fails to present a negative Poisson’s effect, resulting in inefficient energy distribution throughout the structure. As a result, despite having a larger relative density, the non-auxetic RD structure not only provides superior strength but also outperforms the auxetic RE structure in terms of energy absorption under compressive loading. Furthermore, most lattice structures, particularly RD5, demonstrate extraordinary improvements in loading and energy absorption capacities compared with foam material. However, the solid PU sample still surpasses the 3D-printed lattice in terms of loading capacity and energy absorption.

### 3.2. Impact Force Reduction

**Figure 6:**
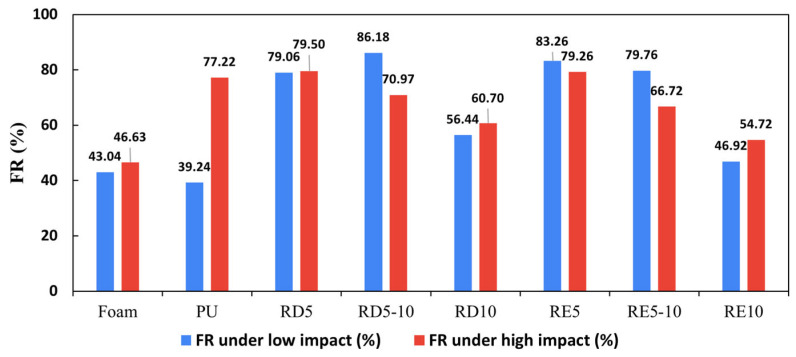
Force reduction rates of all specimens under low- and high-impact conditions.

**Figure 7:**
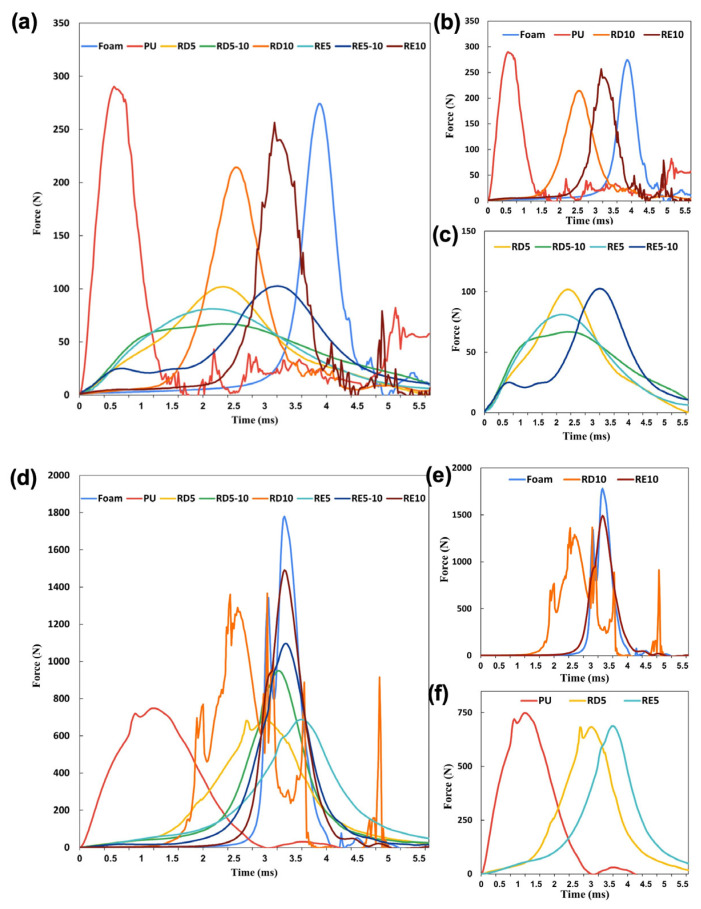
Comparison of force–time curve of (**a**) all specimens, (**b**) specimens with a sharp peak, and (**c**) specimens with a flattened peak in low-impact conditions. Comparison of force–time curve of (**d**) all specimens, (**e**) specimens with a sharp peak, and (**f**) specimens with a flattened peak in high-impact conditions.

The results of the FR rate of the six lattice structures are shown in Table 4 and Figure 6, with Figure 7 illustrating the force–time curves for various cell sizes and structures, along with conventional foam and PU. Across all tests, the RD structures slightly outperform the RE structures, with improvements ranging from 0.3% to 18%. In low-impact conditions, RD5-10 and RE5 structures demonstrate superior FR rates, whereas in high-impact conditions, RD5 and RE5 perform the best. The results indicated that both RD and RE lattices can be optimised for specific impact scenarios, with RD structures generally providing enhanced force reduction performance. On the other hand, RD5 and RE5 structures exhibit better force reduction capabilities in both impact scenarios compared with conventional foam and PU materials.

In low-impact conditions, all six lattice samples show a higher FR rate than the foam and PU sample. The FR rate increases substantially by 2.6 to 3.1 times as the size of the unit cell is reduced from 10 × 10 × 10 mm to 5 × 5 × 10 mm. However, when the cell size is further reduced to 5 × 5 × 5 mm, the rate of enhancement diminishes, and the FR rate of RD5 becomes lower than that of RD5-10 (Figure 6). Under low-impact force, the lattice typically dissipates energy via elastic deformation [46]. A longer impact duration generally corresponds to better energy absorption characteristics. Among the test samples, RD5-10 and RE5 demonstrated broader and more flattened force–time curves, indicating the best performance in terms of force reduction (Figure 7c).

In contrast to the compression test, which applies forces uniformly across the surface of the samples, the ball drop test involves a dynamic and concentrated load at the point of contact [47]. This concentrated load minimises the risk of buckling or structural shifts due to the geometrical imperfections of 3D printing, allowing the RE structure to fully deform for enhanced energy absorption. Despite having different cell structures, RD5-10 and RE5, which have similar relative densities, show the broadest impact duration among all samples. Conversely, RD10 and RE10 exhibit force profiles similar to those of commercial PU and foam (Figure 7b), with narrow peaks that reduce impact force by only 39–56%. In addition, fluctuations in the parameters were observed among PU and RE10, which may result from the rebound of the testing ball and frictional effects, such as rolling or sliding at the interface between the ball and the sample surface. These effects are more pronounced in rigid PU samples and in lattice structures with larger cell sizes (e.g., RE10), which are more susceptible to local buckling or instability under impact. Structures with a higher relative density tend to absorb less energy due to their stiffness, while those with a low relative density may fail to maintain shape and rigidity to withstand the applied load. As a result, the optimal relative density for low-impact force reduction is around 23–25%.

For high-impact conditions, reducing cell size improves force reduction performance. Both RD5 and RE5 absorbed over 79% of impact energy, which is slightly more than the rigid PU plate. A smaller cell size indicates shorter struts, which resist buckling and provide increased stiffness and strength to withstand concentrated impacts. As a result, lattice structures with smaller cell sizes are ideal for high-impact protection, such as in contact and combat sports. Despite variations in their structure and relative density, RD5 and RE5 show only a slight difference in the force reduction rate (79.5% and 79.26%, respectively) and demonstrate broader and flattened force–time curves (Figure 7f). The optimal relative density for high-impact force reduction is believed to be around 25–31%.

In summary, the RD structures demonstrate superior force reduction performance under both low- and high-impact conditions, showing a 13-43% improvement over conventional foam materials, while that of RE structures is about 4-40%. The bending-dominated RD lattices are more effective in terms of energy absorption and load distribution compared with the RE structure. Both RD5 and RE5 structures have excellent impact resistance due to their higher force reduction rates and lower peak forces in both low- and high-impact scenarios, offering better protection for athletes against sudden impacts and collisions.

### 3.3. Bending Rigidity

**Figure 8:**
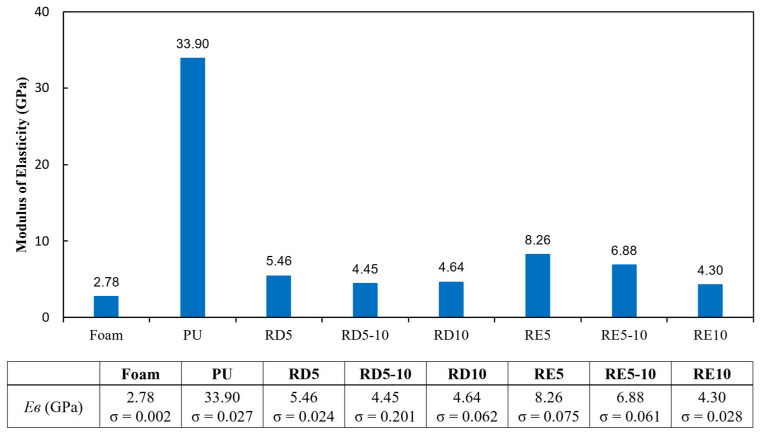
Modulus of elasticity of all specimens.

**Figure 9:**
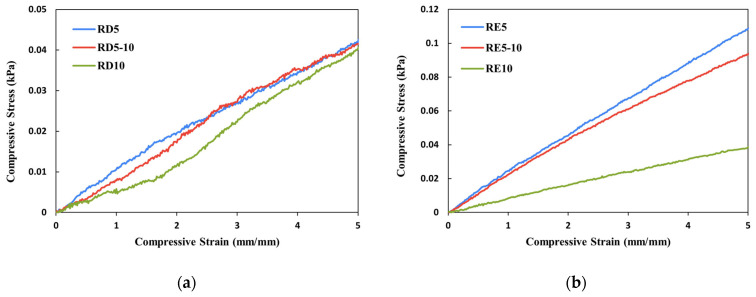
Compressive stress–strain response of (**a**) RD and (**b**) RE specimens.

The experimental results of the bending modulus of elasticity are shown in Figure 8. No fracture or failure was observed in any of the samples, but there was a significant difference in the modulus of elasticity between the six lattice samples and the solid PU material. With the exception of RE10, the stiffness of the RE structures was approximately 40% higher than that of the RD structures. Figure 9 shows the effect of cell sizes on the maximum bending strength of RD and RE structures. As cell size decreases, the rigidity and maximum bending load of the RE structure increase. In contrast, the RD structures show no significant change in their modulus of elasticity across the different cell sizes, indicating that unit cell size has a negligible effect. These findings align with those of Horn et al. [13], who showed no correlation between flexural modulus and strut size in RD structures with a low relative density (below 40%). Interestingly, RE structures with a lower relative density show a higher modulus of elasticity than RD structures. This can be attributed to the geometric differences between bending and stretch-dominant structures. The RE structure, characterised by inward-curving struts, benefits from the auxetic effect, allowing for the upper surface to shrink and the lower surface to expand under load. This results in more efficient load transfer and less bending deformation [48]. In contrast, the RD structure distributes bending loads through bending deformations, with stresses concentrating at the node regions due to interconnected struts, leading to larger deflections and lower bending rigidity [19].

Compared with conventional materials, PU is 4.1 to 7.9 times harder than the proposed lattice structures. Conventional protective equipment, typically made via moulding, lacks conformity with different body shapes due to its stiffness. Lattice materials, however, offer improved flexural performance, though they are only 1.5 to 3 times stiffer than foam. The relatively lower modulus of elasticity in lattice structures enhances shape conformity, providing better fit and comfort. As a result, RD structures with their superior force reduction and flexural properties are well-suited to meet the fitting and impact-resisting requirements of sports protective equipment.

## 4. Conclusions

In this study, we systematically explored the effects of unit cell sizes in bending-dominated rhombic dodecahedron (RD) and stretch-dominated re-entrant (RE) lattice structures, which were produced using DLP printing technology. These structures were compared with conventional foam and rigid PU materials in terms of their compressive strength, energy absorption, impact force reduction, and bending flexural performance. The key findings of this study are summarised below:

1. The RD structure exhibited significantly greater compressive strength and energy absorption capacity than the RE structure, with improvements ranging from 167% to 494%. The RD structure demonstrated uniform deformation under increasing loading, while the RE structure showed buckling and shifting without a negative Poisson’s effect. Both structures, however, had compressive strengths that were approximately 12.9 to 18 times higher than those of conventional foam material.
2. In low-impact tests, RD5-10 and RE5 exhibited superior force reduction performance compared with conventional foam and PU, with enhancements of up to 2.2 and 2 times, respectively. Under high-impact conditions, only RD5 and RE5 performed slightly better than PU, achieving a 1.7 times improvement over foam.
3. The RE structure exhibited greater flexural stiffness than the RD structure. No correlation was found between unit cell size, relative density, and flexural performance in the RD structure, whereas the rigidity of the RE structure increased as cell size decreased. Both structures showed significant flexibility improvements over rigid PU.

The experimental results highlight the superior force reduction and flexural performance of RD and RE structures compared with conventional foam and PU materials. Although PU has higher compressive strength, lattice structures offer a balance between flexural performance and impact reduction ability, which is believed to provide enhanced comfort and protection for wearers. This suggests that there are potential advantages to using lattice structures in sports protection equipment, which require high energy absorption and conformity, and should be lightweight. However, geometrical imperfections in additive manufacturing remain a challenge. Future research should focus on optimising lattice geometry to fit 3D curvatures, such as over the breasts, and evaluate the practical performance of 3D-printed lattice protective gear in terms of wearing comfort and impact resistance.

## Figures and Tables

**Table 1:** Lattice specimen details.

Cell Structure	Sample	Unit Cell Size (L × W × H) (mm)	Mass of 40 × 40 × 40 mm^3^ Cubic (g)	Relative Density (%)
Rhombic dodecahedron (RD)	RD5	5 × 5 × 5 a = 1	20.33	31.45
	RD5-10	5 × 5 × 10 a = 2	15.21	23.53
	RD10	10 × 10 × 10 a = 1	6.03	9.32
Re-entrant (RE)	RE5	5 × 5 × 5 a = 1	16.16	25.00
	RE5-10	5 × 5 × 10 a = 2	12.07	18.67
	RE10	10 × 10 × 10 a = 1	4.58	7.08

**Table 2:** Mechanical properties of printing material (EM+23).

Tensile Strength (MPa)	Elongation at Break (%)	Tensile Modulus (MPa)	Tear Strength (kN/m)
21.59	560	5.79	22.38

**Table 3:** Maximum compressive stress and energy absorption capacity at 40% strain of all specimens.

Parameter	Market Sample		Non-Auxetic			Auxetic		
	Foam	PU	RD5	RD5-10	RD10	RE5	R5-10	RE10
Max. Compressive Stress (kPa)	6.66	4083.20	260.05	120.46	10.46	86.12	24.36	1.67
*Standard Deviation*	*σ = 0.43*	*σ = 33.86*	*σ = 6.71*	*σ = 0.84*	*σ = 0.04*	*σ = 0.08*	*σ = 0.29*	*σ = 0.24*
Energy Absorption Capacity (J/kg)	20.55	532.81	151.32	109.73	21.36	54.79	22.23	5.30
*Standard Deviation*	*σ = 0.016*	*σ = 0.067*	*σ = 0.092*	*σ = 0.076*	*σ = 0.002*	*σ = 0.0002*	*σ = 0.003*	*σ = 0.005*
Poisson’s Ratio	/	/	0.16	0.22	0.50	0.50	-0.42	-1.27
*Standard Deviation*	*/*	*/*	*σ = 0.08*	*σ = 0.33*	*σ = 0.10*	*σ = 0.42*	*σ = 0.90*	*σ = 0.58*

**Table 4:** Mean impact force and force reduction rate of all specimens under low- and high-impact conditions.

		Market Sample		Non-Auxetic			Auxetic		
		Foam	PU	RD5	RD5-10	RD10	RE5	R5-10	RE10
Low impact (500N)	Impact force (N)	275.96	294.36	101.43	66.93	211.04	81.11	98.07	257.17
	*Standard deviation*	*σ = 26.04*	*σ = 7.11*	*σ = 1.75*	*σ = 1.13*	*σ = 7.10*	*σ = 1.11*	*σ = 5.59*	*σ = 15.66*
	FR (%)	43.04	39.24	79.06	86.18	56.44	83.26	79.76	46.92
High impact (3300N)	Impact force (N)	1759.35	750.95	675.76	956.91	1295.39	683.52	1096.95	1492.44
	*Standard deviation*	*σ = 28.35*	*σ = 13.41*	*σ = 20.51*	*σ = 13.10*	*σ = 49.50*	*σ = 14.86*	*σ = 28.21*	*σ = 20.67*
	FR (%)	46.63	77.22	79.50	70.97	60.70	79.26	66.72	54.72

## Data Availability

The original contributions presented in this study are included in the article. Further inquiries can be directed to the corresponding author.
